# The arginine methyltransferase *Carm1* is necessary for heart development

**DOI:** 10.1093/g3journal/jkac155

**Published:** 2022-06-23

**Authors:** Sophie Jamet, Seungshin Ha, Tzu-Hua Ho, Scott Houghtaling, Andrew Timms, Kai Yu, Alison Paquette, Ali Murat Maga, Nicholas D E Greene, David R Beier

**Affiliations:** Center for Developmental Biology and Regenerative Medicine, Seattle Children’s Research Institute, Seattle, WA 98101, USA; Center for Developmental Biology and Regenerative Medicine, Seattle Children’s Research Institute, Seattle, WA 98101, USA; Center for Developmental Biology and Regenerative Medicine, Seattle Children’s Research Institute, Seattle, WA 98101, USA; Center for Developmental Biology and Regenerative Medicine, Seattle Children’s Research Institute, Seattle, WA 98101, USA; Center for Developmental Biology and Regenerative Medicine, Seattle Children’s Research Institute, Seattle, WA 98101, USA; Center for Developmental Biology and Regenerative Medicine, Seattle Children’s Research Institute, Seattle, WA 98101, USA; Department of Pediatrics, University of Washington School of Medicine, Seattle, WA 98195, USA; Center for Developmental Biology and Regenerative Medicine, Seattle Children’s Research Institute, Seattle, WA 98101, USA; Department of Pediatrics, University of Washington School of Medicine, Seattle, WA 98195, USA; Center for Developmental Biology and Regenerative Medicine, Seattle Children’s Research Institute, Seattle, WA 98101, USA; Department of Pediatrics, University of Washington School of Medicine, Seattle, WA 98195, USA; Developmental Biology & Cancer Department, UCL Great Ormond Street Institute of Child Health, London WC1N 1EH, UK; Center for Developmental Biology and Regenerative Medicine, Seattle Children’s Research Institute, Seattle, WA 98101, USA; Department of Pediatrics, University of Washington School of Medicine, Seattle, WA 98195, USA

**Keywords:** ENU mutagenesis, *Carm1*, heart development, *Pax3*

## Abstract

To discover genes implicated in human congenital disorders, we performed ENU mutagenesis in the mouse and screened for mutations affecting embryonic development. In this work, we report defects of heart development in mice homozygous for a mutation of coactivator-associated arginine methyltransferase 1 (*Carm1*). While *Carm1* has been extensively studied, it has never been previously associated with a role in heart development. Phenotype analysis combining histology and microcomputed tomography imaging shows a range of cardiac defects. Most notably, many affected midgestation embryos appear to have cardiac rupture and hemorrhaging in the thorax. Mice that survive to late gestation show a variety of cardiac defects, including ventricular septal defects, double outlet right ventricle, and persistent truncus arteriosus. Transcriptome analyses of the mutant embryos by mRNA-seq reveal the perturbation of several genes involved in cardiac morphogenesis and muscle development and function. In addition, we observe the mislocalization of cardiac neural crest cells at E12.5 in the outflow tract. The cardiac phenotype of *Carm1* mutant embryos is similar to that of *Pax3* null mutants, and PAX3 is a putative target of CARM1. However, our analysis does not support the hypothesis that developmental defects in *Carm1* mutant embryos are primarily due to a functional defect of PAX3.

## Introduction

Congenital heart defects (CHDs) are the most common of human congenital anomalies, affecting 9.1 per 1,000 liveborn infants (van der Linde *et al.* 2011). CHDs are estimated to have caused the death of 3.5% of children under 5 years old worldwide in 2019 (Global Burden of Disease Collaborative Network; http://www.healthdata.org/gbd/2019). Over recent years, genomic analysis has uncovered many genes that, when dysregulated, cause cardiac defects (reviewed in Pierpont *et al.* 2018), but 70–80% of CHDs remain unexplained by either genetic or environmental causes (Cowan and Ware 2015). The discovery of the genetic basis of CHDs will benefit patient care and genetic counseling of families.

To discover new genes not previously associated with congenital anomalies, we have carried out several mutagenesis screens in the mouse for recessive mutations that cause defects in organogenesis (Moran *et al.* 2006; Kamp *et al.* 2010; Dwyer *et al.* 2011; Stottmann *et al.* 2011; Gallego-Llamas *et al.* 2015; Ha *et al.* 2015; Geister *et al.* 2018). This strategy allows us to discover new genes implicated in developmental phenotypes in an unbiased fashion. In this report, we describe a mutant line with a striking midgestation phenotype, in which a substantial number of mutant embryos show blood in the thoracic cavity, suggesting rupture of the heart or blood vessels. Mutant embryos fail to properly form separation between the ventricles of the heart (ventricular septal defects, VSDs). The formation of the blood vessels connecting the heart is also compromised, with the aorta connecting the wrong chamber (double outlet right ventricle, DORV) or fused with the pulmonary artery (persistent truncus arteriosus, PTA). No mutants survive to weaning age.

In the mouse, cardiac progenitor cells (CPCs) start to differentiate at E7.5, expressing the marker *Mesp1* and forming the cardiac crescent (reviewed in Meilhac *et al.* 2014). Later, at E8.25, CPCs form 2 distinct populations: the first and second heart fields. The first heart field forms a linear tube. Cells from the second heart field migrate at the anterior and posterior poles of the tube. At E9.25, the tube loops and cells from the second heart field start to form the right ventricle. Cells from the first heart field form the left ventricle. Both lineages participate in the formation of the atria and other parts of the heart. In addition, cardiac neural crest cells (NCCs) migrate from the neural tube and populate the pharyngeal arches and the anterior opening of the right ventricle called the outflow tract (OFT) at E10.5. Here, they contribute to the formation and the separation of the aorta and pulmonary artery, separating the systemic and pulmonary circulation. At the same time, a constriction between the left ventricle and the atria forms called the atrioventricular canal.

The mutation we identified affects the gene coding for the coactivator-associated methyltransferase 1 (*Carm1*), also known as protein arginine methyltransferase 4 (*Prmt4*). The methylation of many proteins is a regulated post-translational modification that is required for their function (Bedford and Clarke 2009). Protein arginine methyltransferases (PRMTs) are enzymes that transfer methyl groups onto arginine residues. *Carm1* is a transcriptional coactivator whose activity is mediated through methylation of histones and/or other proteins of the initiation complex (Lee *et al.* 2002); *Carm1* is known to have numerous other protein targets as well (Bedford and Clarke 2009). Furthermore, a variety of roles for *Carm1* in addition to its transcriptional coactivator function have been described (reviewed in Suresh *et al.* 2021). The knockout of *Carm1* in the mouse is known to affect several aspects of embryonic development, including maintenance of pluripotency in the early embryo (Torres-Padilla *et al.* 2007; Hupalowska *et al.* 2018; Quintero *et al.* 2018), thymus development (Kim *et al.* 2004), ossification (Ito *et al.* 2009), skeletal muscle differentiation (Chen *et al.* 2002; Batut *et al.* 2011; Chang *et al.* 2018), astroglial differentiation (Selvi *et al.* 2015), adipocyte differentiation (Yadav *et al.* 2008), spermiogenesis (Bao *et al.* 2018) and lung development (O’Brien *et al.* 2010), and autophagy in aged or injured adult hearts (Li *et al.* 2017; Wang *et al.* 2019). However, there has been no previous report of a role for *Carm1* in embryonic heart development.

In this work, we investigate the effect of the loss of *Carm1* on several aspects of heart development. We describe the phenotype observed in embryonic hearts of *Carm1* mutants by histology and also by 3D imaging using microcomputed tomography (micro-CT). mRNA sequencing was used to identify genes differentially expressed in *Carm1* mutants at E12.5. Gene set enrichment analysis shows enrichment in genes involved in already described aspects of *Carm1* activity such as muscle development and function, as well as new ones, including heart morphogenesis. We show that a failure of *Carm1* activity affects the expression of genes known to be involved in heart development. Interestingly, we show the mislocalization of cardiac NCCs in the OFT. Finally, we explored a possible role of *Carm1* in regulating the activity of the transcription factor *Pax3*, which is involved in cardiac NCC differentiation.

## Methods

### Mice

The generation of *Carm1^A296E^* (MGI:5505489) using ENU mutagenesis was previously described (Ha *et al.* 2015), where it was designated “line 8.” The mutant line originated from mutagenized A/J (Jackson Laboratory) and is maintained by serial backcrosses to C57BL/6J females. Genotyping is done by sequencing the PCR product generated using the primers DB915_108f: AGAGGCCATGGACAAACAAC and DB916_108r: AGGCGGAGTTCTGAGTTCAA. *Carm1^tm1Mtb^* knockout mice were kindly provided by Dr. Mark Bedford (Yadav *et al.* 2003). Animals were maintained in accordance with guidelines of the National Institutes of Health and the Seattle Children’s Research Institute’s Institutional Animal Care and Use Committee.

Husbandry of mice carrying the *Pax3^Sp2H^* mutation was done in the Greene lab. These mice have been maintained as a closed colony for more than 50 generations on a mixed background derived from CBA/Ca, 101, and C3H/He. Animal studies were carried out under regulations of the Animals (Scientific Procedures) Act 1986 of the UK Government, and in accordance with the guidance issued by the Medical Research Council, UK, in Responsibility in the Use of Animals for Medical Research (July 1993).

### Mouse embryonic fibroblasts

Primary mouse embryonic fibroblasts (MEFs) were isolated from individual E13.5 embryos by enzymatic digestion using 0.25% trypsin-EDTA. MEFs were immortalized by transfection with the SV40 large T-antigen expression plasmid SV40 1: pBSSVD2005, a gift from David Ron (Addgene plasmid # 21826; RRID:Addgene_21826), using a protocol from Heather P. Harding (revised 2003 May 11) (https://media.addgene.org/data/45/42/165f51de-af64-11e0-90fe-003048dd6500.pdf).

### Histology

Tissues were collected from embryos at various embryonic timepoints and fixed in Bouin’s solution. They were dehydrated in ethanol to 100%, and then in xylene, and embedded in paraffin. Sections were then cut at a thickness of 10 µm and mounted on slides. The sections were stained using Masson’s Trichrome Staining kit from Poly Scientific R&D Corp following the manufacturer’s instruction. The sections were deparaffinized, hydrated, and fixed again in Bouin’s fixative for 1 h at a 56°C and rinsed in water. Then, they were stained with hematoxylin for 10 min, scarlet-acid fuchsin for 2 min, phosphotungstic and phosphomolymbic acid for 15 min, aniline blue for 5 min, and then differentiated in 1% glacial acetic acid for 3 min. The sections were then dehydrated and glass coverslips were mounted. The stained sections were examined using Leica bright-field microscope. In situ hybridizations were done as described in Lufkin (2007). The probes used were synthesized from the following plasmids: pISH-Isl1 was a gift from Peter Mombaerts (Addgene plasmid # 84311; RRID:Addgene_84311); mFGF8b was a gift from Gail Martin (Addgene plasmid # 22090; RRID:Addgene_22090); and pBS-mSema3C was a gift from Jonathan Raper (Addgene plasmid # 16426;RRID: Addgene_16426).

### Immunohistochemistry

Embryos were dissected, fixed in 4% PFA in PBS overnight, incubated in 30% sucrose overnight, and embedded in OCT freezing medium on dry ice/methylbutane slurry. The OCT-embedded embryos were cryosectioned at a thickness of 5 µm, mounted on glass slides. The sections were washed with PBS, incubated with blocking solution (3% goat serum, 3% bovine serum albumin, 0.3% Triton X-100 in PBS) for 2 h, with the primary antibodies for overnight at 4°C, with the Alexa Fluor-conjugated secondary antibodies for 2 h at room temperature, and then with Hoechst for 5 min. The primary antibodies used are mouse anti-CARM1 antibodies (Cell Signaling Technologies, #12495; RRID:AB_2797935), anti-CARM1 monoclonal antibody, PRMT4/CARM1 C31G9 Rabbit mAb #3379 from Cell Signaling Technology (RRID:AB_2068433), or anti-PAX3 monoclonal antibody from the Developmental Studies Hybridoma Bank (DSHB; RRID:AB_528426, deposited by C.P. Ordahl). The immunostained sections were imaged using Leica confocal microscope.

### Immunoprecipitation and western blot

Embryonic brains were collected at E16.5 and homogenized in lysis buffer (20 mM Tris-HCl, 150 mM NaCl, 0.1% SDS, 1% Triton X-100, 1% sodium deoxycholate, 1 mM PMSF, 1X protease, and phosphatase inhibitor cocktail). The samples were centrifuged to collect the supernatant. BCA protein assay was performed to measure protein concentration in the supernatant. Approximately 2 mg of protein was used for immunoprecipitation using 1 µl of mouse anti-CARM1 antibodies (Cell Signaling Technologies, #12495; RRID:AB_2797935), which were crosslinked to PureProteome Protein A/G mix magnetic beads (Millipore). The eluted samples were separated on SDS-PAGE gel and transferred onto a PVDF membrane. The membrane was incubated with blocking solution (5% milk, 1X TBS, 0.1% Tween-20) for 2 h, with mouse anti-CARM1 antibodies (1:2,000) for overnight at 4°C, then with HRP-conjugated antimouse secondary antibodies for 2 h at room temperature. SuperSignal West Femto substrate (Thermo Fisher Scientific) was used for detection. The lysates were also analyzed by western blotting using anti-GAPDH (Cell Signaling Technologies, #5174, RRID:AB_10622025; 1:2,000) to show the same amount of protein was used for IP.

### Micro-CT analysis

E18.5 hearts were dissected and fixed for 72 h in 4% PFA. Hearts were stained for 72 h in the 2% phosphotungstic acid, 0.1% hematoxylin (PTAH). PTAH stained hearts were imaged using Skyscan micro-CT set for the following scanning mode: 18 micron, AI 0.5 mm, 360 ms, 55 kV, 180 µA, 3,000 HU, 0.70°C. Scans were reconstructed using NRecon software with the following settings: misalignment between −2 and +2, dynamic range mode, min 0.01, max 0.2. Reconstructed slices were imported and visualized using the SlicerMorph extension (Rolfe *et al.* 2021) of the 3D Slicer biomedical visualization program.

### Expression analysis

Timed matings were set up using intercrosses of heterozygous *Carm1^tm1Mtb^* or *Pax3^Sp2h^* and harvesting embryos at embryonic day 12.5 (E12.5). Mice were genotyped and RNA was isolated from homogenized whole embryos using the PureLink RNA Mini Kit (Invitrogen). RNA quality was assessed using an Agilent 4200 Tapestation and samples with an RNA Integrity Number equivalent (RINe) >9 were selected. RNA library preparation with polyA enrichment and paired-end 150 bp sequencing was performed by the commercial provider Novogene Corporation, Inc. using the Illumina NovaSeq platform. RNA sequence reads were aligned to the mouse genome (mm10) using Star (Dobin *et al.* 2013). Aligned reads mapping to the exons of a gene were quantitated with featureCounts (Liao *et al.* 2014). DESeq2 (Love *et al.* 2014) was used to normalize the data and transform the data using variance-stabilizating transformation, examine the relationship between samples, and to look for differential expression between groups of samples. For all analyses, genes were considered statistically significant with a false discovery rate (FDR) <0.05 using the Benjamini–Hochberg method (Benjamini and Hochberg 1995). Pathway enrichment was performed using Gene Set Enrichment Analysis (GSEA); which performs permutation testing (*N* = 10,000 permutations) to evaluate if the differentially expressed genes (ranked by *P*-value) appear in specific pathways more than would be expected by chance (Subramanian *et al.* 2005). For this analysis, we included all Gene Ontology Biological pathways (GO:BP). GO:BP terms were summarized and condensed using semantic similarities measures implemented within REVIGO (Supek *et al.* 2011) and visualized in cytoscape. Overrepresentation analysis was performed on concordant genes using fishers exact tests implemented in PANTHER (Mi *et al.* 2013) with the background list of all genes included in both analyses.

### Patient sequence variant analysis

The results analyzed and shown here are based upon data generated by Gabriella Miller Kids First Pediatric Research Program (projects phs001138.v3.p2 and phs001735.v2.p1) and were accessed from dbGaP (www.ncbi.nlm.nih.gov/gap). For phs001138.v3.p2, we downloaded a VCF that had been annotated by VEP for each pedigree. We used Slivar (version 0.2.1) to filter variants based on inheritance pattern, population frequency, quality, coverage, allele balance, and impact on the gene. We required a genotype quality of >15, coverage >10 in all family members, an allele balance of >0.2 for heterozygous variants, and a <0.01 frequency in any gnomAD version 3 population. We identified all variants that passed those filters and de novo variants (heterozygous in child and homozygous reference in both parents). For phs001735.v2.p1, we did not have complete family structures and a cohort-based VCF. Therefore, we subset all affected individuals using bcftools (version 1.11) and annotated using annovar. We prioritized variants that were exonic (excluding synonymous variants) or within a splice site (±2 bp from exon) and had a frequency of <0.01 in gnomAD version 3. To determine the inheritance pattern of the identified variants in CARM1, we used a supplied IBD kinship analysis to identify the parents of the affected individuals and examined their genotypes.

*Carm1^A296E^* mice are available upon request and will be submitted to the Mutant Mouse Resource and Research Center (MMRRC) repository. Gene expression data are available at GEO with the accession number: GSE193588.

## Results

### ENU mutagenesis screen and Identification of the mutation in *Carm1*

**Fig. 1.**
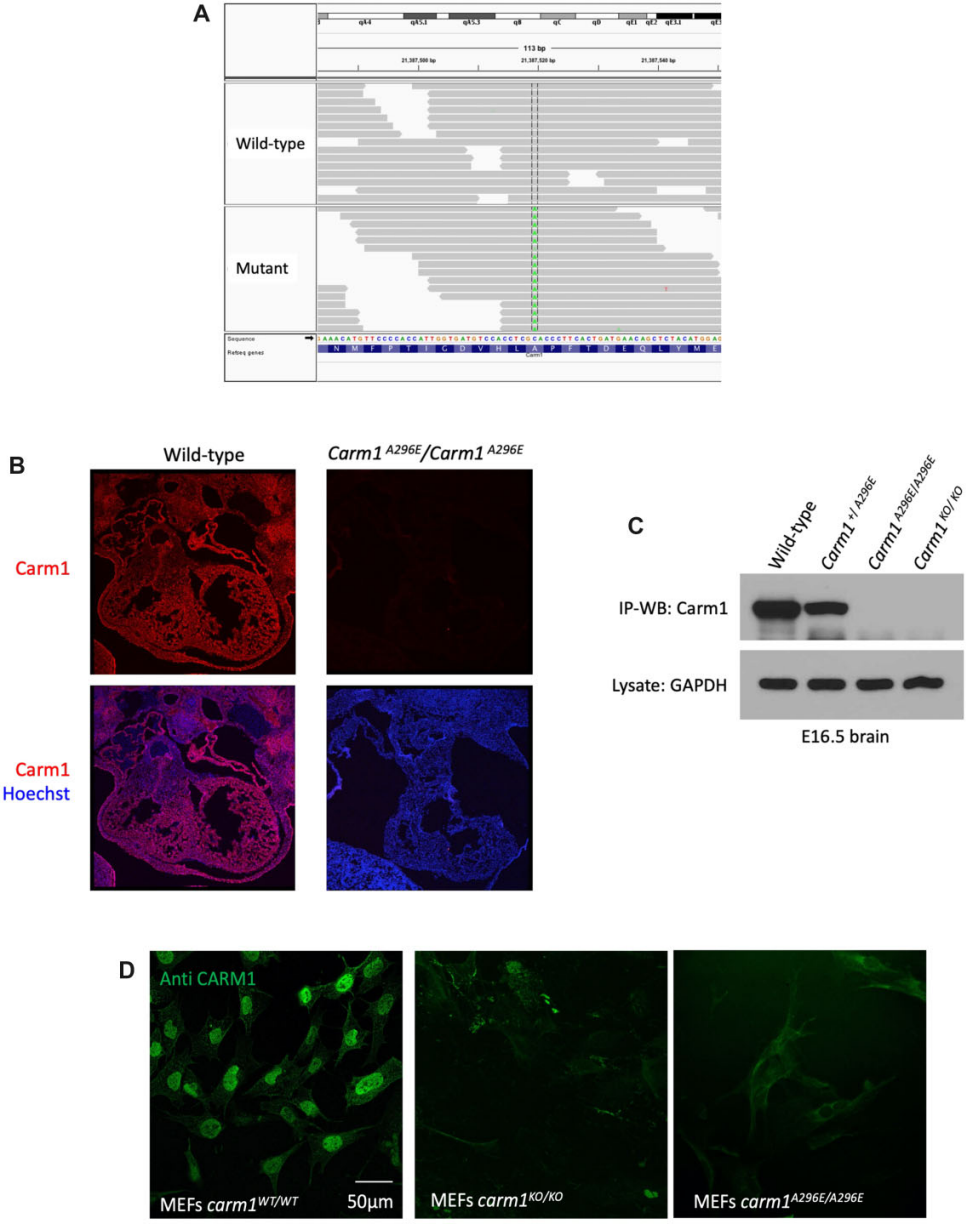
Discovery of *Carm1^A296E^* mutation and analysis of CARM1 expression. a) Sequence analysis identified a mutation of a C to an A at chr9:21583075 (mm10), which is in exon 7 of the gene encoding *Carm1.* b) CARM1 protein is not detectable in heart tissue from *Carm1^A296E^* mutant mice. c) Immunoprecipitation/western analysis demonstrates that CARM1 protein is not present in tissue from *Carm1^A296E^* and *Carm1^KO^* mutant mice. d) CARM1 protein is readily detected in nuclei in MEFs from wild-type embryos, but is not expressed in MEFs from *Carm1^A296E^* and *Carm1^KO^* mutant mice.

In a screen of ENU-mutagenized mice at E18.5 pursued to identify mice with neurodevelopmental defects, we identified a mutant originally called “line 8” with cortical heterotopias, smaller embryo size, and variable omphalocele, abnormal limb morphology, abnormal craniofacial morphology, and cleft palate (Ha *et al.* 2015). Despite the variable phenotype, genetic mapping analysis located the mutation to a region of proximal chromosome 9. Sequence analysis revealed this line carried a mutation of a C to an A at chr9:21583075 (mm10), which is in exon 7 of the gene encoding *Carm1* (Fig. 1a). The mutation is nonsynonymous, resulting in an alanine residue being replaced by a glutamic acid residue at position 296. This mutation is predicted to be probably damaging with a score of 0.99 using PolyPhen (Adzhubei *et al.* 2010). The affected residue is in the catalytic domain of the protein (amino acids 147–454, SAM-dependent methyltransferase PRMT-type domain). The alignment shows the residue belongs to a highly conserved region of the protein (Supplementary Fig. 1).

To assess whether this missense mutation, now called *Carm1^A296E^*, was causal for the observed organogenesis defects, we tested it for noncomplementation using the *Carm1* null mutant *Carm1^tm1Mtb^* (called here *Carm1^KO^*; Yadav *et al.* 2003). Compound *Carm1^A296E/KO^* embryos examined at E18.5 had the same gross anomalies as *Carm1^A296E^* homozygous mutants (Supplementary Fig. 2), and no survival was observed postnatally (Table 1), proving that the A296E mutation we identified is causal for the abnormal phenotype in line 8. As discussed below, *Carm1^KO^* mutant embryos have the same variably expressed cardiac defects as we observed in *Carm1^A296E^*.

**Table 1.** Analysis of *Carm1^A296E^* or *Carm1^KO^* mutant recovery during embryonic development, and complementation analysis of the *Carm1^A296^* and *Carm1^KO^* mutants.

A	*Carm1* allele			E18.5	21 d
	A296E/+			22	34
	A296E/A296E			5	0
	+/+			17	17
	Litters			6	8
	% A296E/A296E			11%	0%
	*P*-value (Chi^2^)			0.038	0.0002
B	*Carm1* allele	E10.5	E12.5	E18.5	21 d
	KO/+	95	128	28	22
	KO/KO	34	45	19	0
	+/+	56	77	25	14
	Litters	22	21	10	10
	% KO/KO	18%	18%	26%	0%
	*P*-value (Chi^2^)	0.068	0.015	0.10	0.0018
C	*Carm1* allele				21 d
	A296E/+				6
	KO/+				8
	KO/296E				0
	+/+				5
	Litters				3
	% KO/A296E				0%

To evaluate the effect of the *Carm1^A296E^* mutation, we examined CARM1 protein expression in the mutant mice. Strikingly, CARM1 is not evident in the heart in *Carm1^A296E/A296E^* embryos (Fig. 1b), showing the drastic effect of the A296E missense mutation on CARM1 expression. The absence of expression is also evident in western blots of embryonic brain, which were analyzed in the context of the original ascertainment of the ENU-induced mutant as affecting cortical development (Ha *et al.* 2015; Fig. 1c). We also examined protein expression in MEFs. In cells derived from wild-type mice, CARM1 protein is readily detected in nuclei (Fig. 1d). In MEFs from *Carm1^KO/KO^* and *Carm1^A296E/A296E^* embryos, no expression of CARM1 can be observed.

### *Carm1* mutants show a range of cardiac defects

**Fig. 2.**
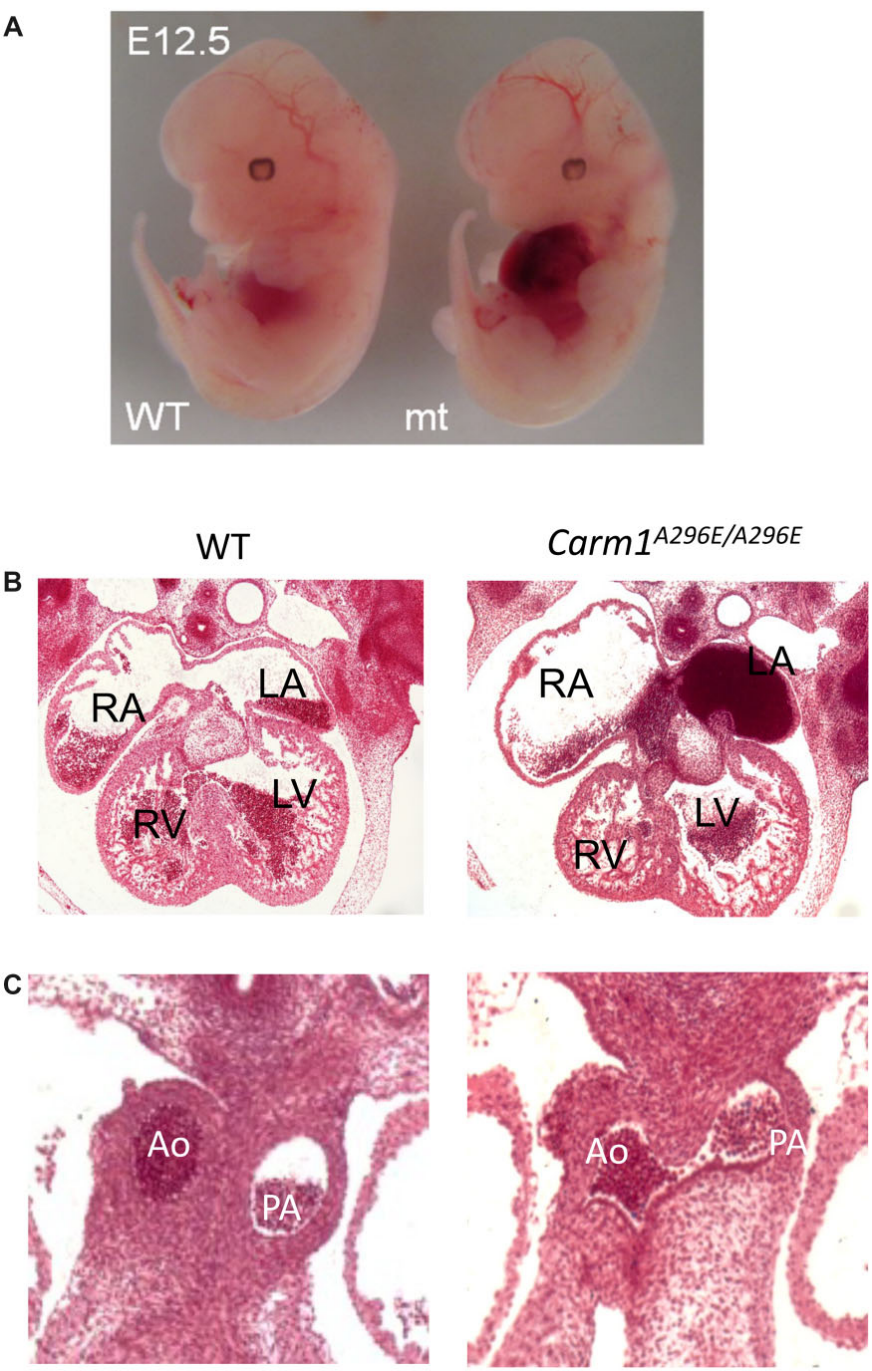
Cardiac defects in *Carm1* mutants at E12.5. a) An E12.5 *Carm1^A296E^* mutant embryo with blood in the thoracic cavity. b) Histology of an E12.5 *Carm1^A296E^* mutant embryo showing apparent atrioventricular obstruction with blood in the atrium. RA, right atrium; RV right ventricle; LA, left atrium; LV, left ventricle. c) Histology of an E12.5 *Carm1^A296E^* mutant embryo showing persistent truncus arteriosus. Ao, aorta; PA, pulmonary artery.

A fortuitous dissection at E12.5 unexpectedly revealed embryos with hemorrhaging in the thoracic cavity (Fig. 2a). Initial genetic studies indicated this phenotype was highly penetrant: of 16 homozygote *Carm1^A296E/A296E^* mutants examined at E12.5, 8 (50%) had a visible heart defect at the time of dissection. The frequency of observing midgestation thoracic bleeding has decreased over time, perhaps because of sequential breeding of the mutation onto C57BL/6J. Evidence for a midgestation cardiac defect is supported by genetic analysis; genotyping of 44 embryos at E18.5 revealed that only 11% were homozygous *Carm1^A296E/296E^* mutants (Table 1), which is fewer than the expected 25%. In their characterization of the *Carm1^KO^* mutant line, Yadav *et al.* (2003) also found a reduction of homozygous mutant mice at E18.5. We did not see this, although we tested a much smaller sample size. Of note, no *Carm1^A296E/296E^* or *Carm1^KO/KO^* mutants have been found to survive to weaning age (Table 1).

**Fig. 3.**
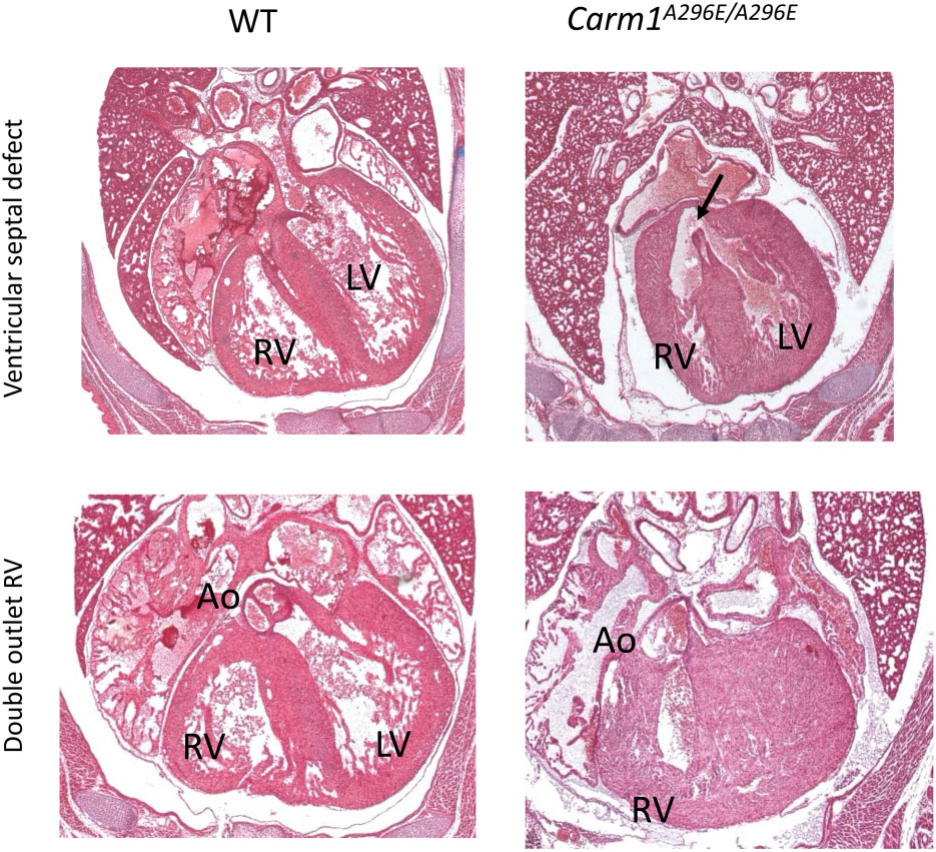
Cardiac defects in *Carm1* mutants at E18.5. a) Histology of an E18.5 *Carm1^A296E^* mutant embryo showing a VSD (arrow). b) Histology of an E18.5 *Carm1^A296E^* mutant embryo showing DORV.

Histological analysis of *Carm1 ^A296E/A296E^* and *Carm1^KO/KO^* embryos show several similar cardiac defects. At E12.5, the cardiac phenotype showed variable expressivity; this included presenting with a smaller left atrium, blood pooling in a left atrium, extra endocardial cushion tissue growing into and obstructing the mitral valve, an enlarged right atrium, or a torn left atrium with blood filling the thoracic cavity (Fig. 2b and Supplementary Fig. 3). A subset of mutants shows communication between the aorta and the pulmonary artery (PTA, Fig. 2c and Supplementary Fig. 3), whereas wild-type embryos already show a separation at the same age. Older embryos (E18.5) show a variety of cardiac defects, including VSDs (Fig. 3a and Supplementary Fig. 3) and DORV (Fig. 3b).

Micro-CT has been shown to be a remarkably accurate tool for the screening of CHD in mouse embryos (Liu *et al.* 2017). Imaging of E18.5 hearts by micro-CT and 3D reconstructions confirm our histological studies; e.g. *Carm1^KO/KO^* E18.5 embryos show VSD (*n* = 3; Supplementary Fig. 3).

### Cardiac NCCs are mislocalized in *Carm1^KO/KO^* mutant mice

**Fig. 4.**
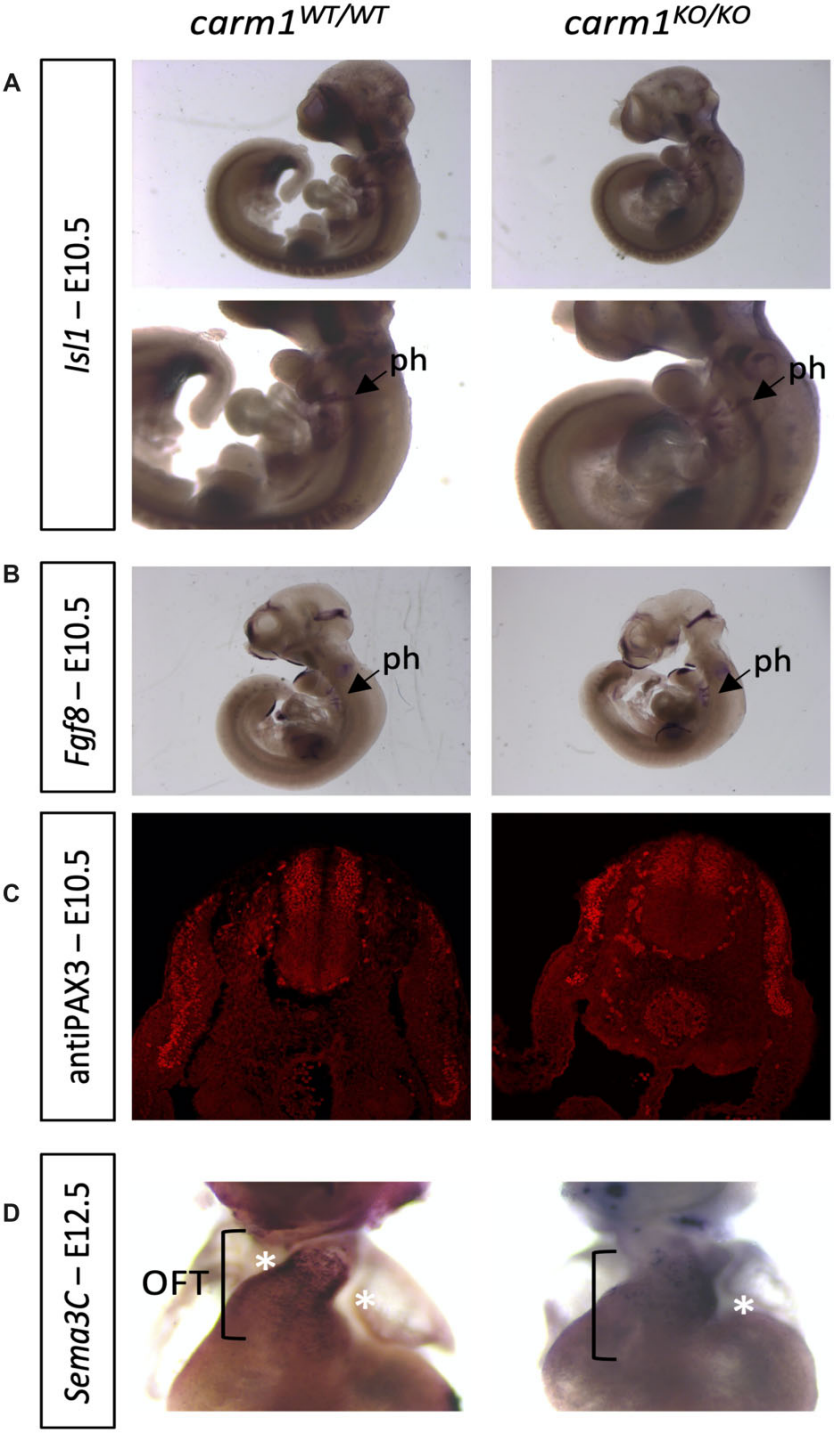
Whole-mount in situ hybridization and immunohistochemistry to characterize CPCs in *Carm1^KO^* embryos. a) There is no difference in the localization of the first heart field marker *Isl1* in the pharyngeal arches of wild-type vs mutant embryos at E10.5. ph, pharyngeal arches. b) There is no difference in the localization of the second heart field marker *Fgf8* in wild-type vs mutant embryos at E10.5. c) PAX3 is normally expressed in the dorsal part of the neural tube at E10.5 in *Carm1^KO^* mutant embryos, suggesting there is proper differentiation of premigratory NCCs. NT, neural tube; gl, ganglion; s, somite; da, dorsal aorta. d) At E12.5 cardiac NCCs expressing *Sema3C* (asterisks) form 2 focal groups of cells in wild-type embryos. In contrast, in *Carm1^KO^* mutant embryos, cells expressing *Sema3C* are dispersed and fail to condense. OFT, outflow tract.

To characterize the localization of cardiac progenitors in *Carm1^KO/KO^* embryos, we performed whole-mount in situ hybridization for the second heart field marker *Isl1*. At 10.5, we see no difference in the localization of expression of *Isl1* in the pharyngeal arches (Fig. 4a).

At the same stage, we investigated the expression of *Fibroblast growth factor 8* (*Fgf8*) by whole-mount in situ hybridization (Fig. 4b). *Fgf8* is critical factor for the development of the OFT; it is expressed by second heart field derived myocardium and its expression is regulated by *Jag1-Notch* signaling from the second heart field (High *et al.* 2009). Hence *Fgf8* is a useful measure of Notch signaling in the second heart field. We observe no difference of *Fgf8* expression in the pharyngeal arches at E10.5 between *Carm^1KO/KO^* and wild-type embryos.

Cardiac NCCs express different markers during their migration from the neural tube to the OFT. In the neural tube, premigratory NCCs express the transcription factor *Pax3*. We detect proper expression of PAX3 in the dorsal part of the neural tube at E10.5 in *Carm1^KO/KO^* mutant embryos (Fig. 4c). This suggests proper differentiation of premigratory NCCs at that stage. When cardiac NCCs localize to the OFT they express the guidance cue *Semaphorin 3C* (*Sema3c*). By in situ hybridization, we detect cells expressing *Sema3C* in the OFT (Fig. 4d). In wild-type embryos, cardiac NCCs expressing *Sema3C* form 2 focal groups of cells, as expected. In contrast, in *Carm1^KO/KO^* mutant embryos, cells expressing *Sema3C* are dispersed and fail to condense.

### Differential gene expression in *Carm1^KO/KO^* mutant embryos

**Fig. 5.**
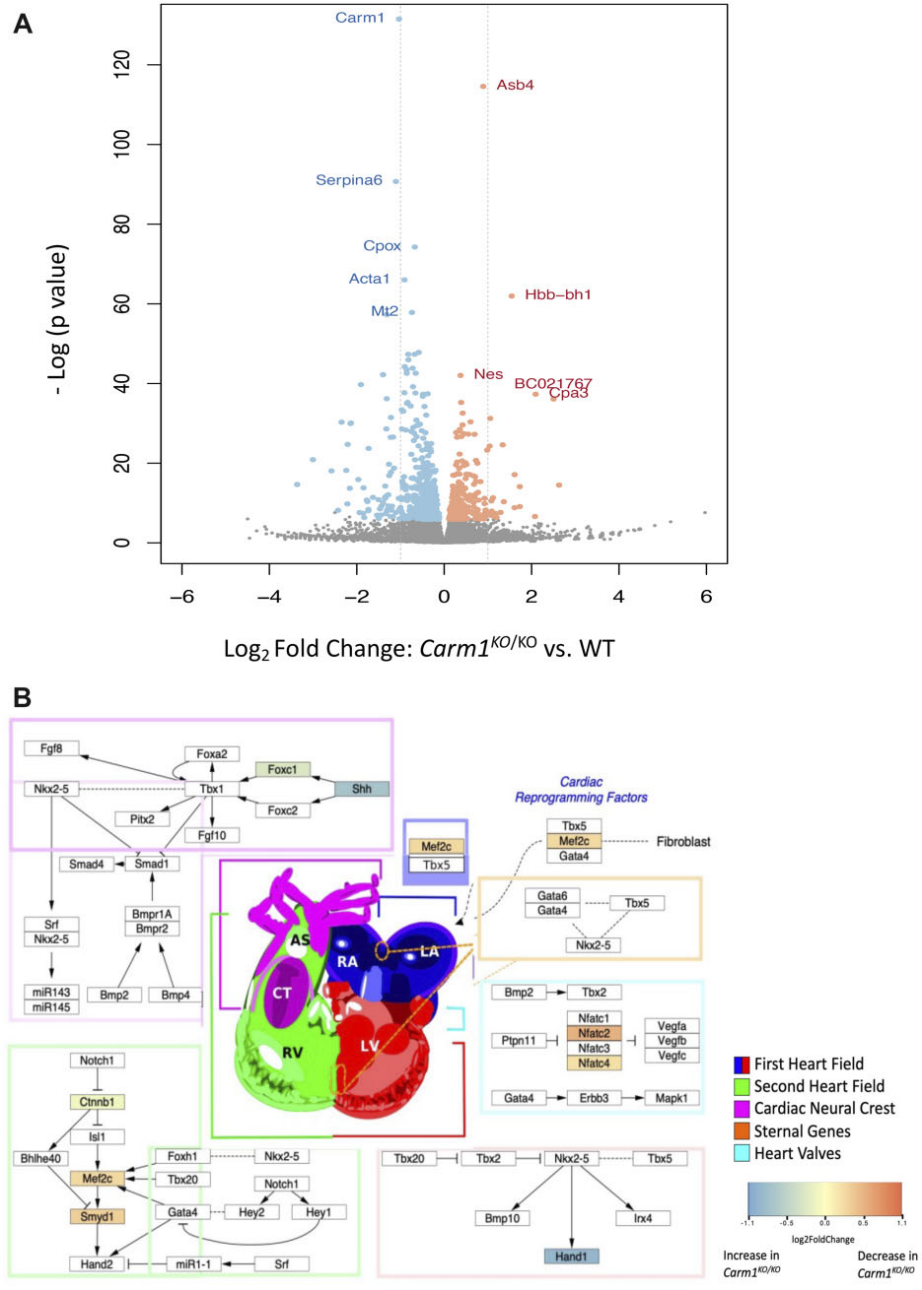
Expression analysis of E12.5 *Carm1^KO^*mutant embryos. a) Eight hundred and sixty genes are differentially expressed (*n* = 3WT and 3KO, *P*_adj_ < 5%). b) Examination of genes known to be involved in heart development (from WikiPathway #2067) reveals both decreased and increased expression in *Carm1^KO^* mutant embryos compared to wild type. Fold changes in expression are indicated for genes with a *P*- adjusted value >0.05 (Supplementary Table 2).

*Carm1* is widely expressed during embryonic organogenesis, including in the developing heart (Supplementary Fig. 5). To explore the effect of the loss of *Carm1* during development, we performed mRNA sequencing of RNA isolated from E12.5 *Carm1^KO/KO^* embryos and wild-type controls (*N* = 3/group). We identified 854 differentially expressed genes (FDR adjusted *P* < 0.05), including 307 that were upregulated in the absence of *Carm1* and 547 that were downregulated (Fig. 5a). We note that *Carm1* gene expression is significantly reduced in the mutants. The *Carm1* KO mutation is a deletion of exons 2 and 3, and the predicted residual transcript that fuses exon 1 and 4 introduces a frameshift. Examination of the RNAseq data confirms this; and the reduction of *Carm1* mRNA is presumably due to nonsense-mediated decay.

GSEA revealed 94 pathways with significant enrichment of genes underexpressed in *Carm1^KO/KO^* samples compared to wild type; these involved inflammation, lipid regulation, skeletal muscle development, and metabolic processes (FDR adjusted *P* < 0.05; Supplementary Table 1). We did not identify any pathways enriched for genes overexpressed in *Carm1^KO/KO^* compared to wild type. The enriched gene sets include those related to several known functions of *Carm1*, including skeletal muscle development. Our data also show enrichment in genes involved in nervous system functions and development, suggesting a role for *Carm1* in neuronal lineage, and consistent with our observation of cortical defects in mutant mice (Ha *et al.* 2015). Importantly, we see enrichment for genes involved in heart function.

Based on changes in heart development observed in *Carm1 ^KO/KO^* mice, we performed a targeted pathway analysis to examine differentially expressed genes known to be involved in heart development using the “Heart Development (*Mus Musculus*)” WikiPathway (#2067; Fig. 5b; Supplementary Table 2). We identified decreased expression (Log Fold Change −0.27, FDR adjusted *P* = 0.003) of the important cardiac transcription factor Myocyte Enhancer Factor 2C (*Mef2c*), a target of ISL1 that is necessary for proper second heart field development (Dodou *et al.* 2004; von Both *et al.* 2004). Its target gene SET and MYND domain-containing protein 1 (*Smyd1*), which is involved in the right ventricle development in mice (Gottlieb *et al.* 2002), is also reduced (log FC −0.3, FDR adjusted *P* = 0.02). Upstream of *Mef2c*, we see an increase of expression (Log FC 0.1, FDR adjusted *P* = 0.04) of the gene *Ctnnb1*, coding for β-catenin. In the second heart field, the Wnt/β-catenin pathway maintains the progenitors in a proliferative and undifferentiated state (Klaus *et al.* 2007; Ruiz-Villalba *et al.* 2016). Also of note, we see an increase in the expression of *Hand1* (Log FC 0.9, FDR adjusted *P* = 0.02) and a decrease of expression of the Nuclear Factor of Activated T-cell (NFAT) family members 2 and 4 (*Nfat2* and *Nfat4*), revealing misregulation of genes involved in the differentiation of progenitors from the first heart field. We also observe an increase in the expression of *Shh* (Log FC 0.8, FDR adjusted *P* = 0.0002) and *Foxc1* (LogFC 0.3, FDR adjusted *P* = 0.005), both involved in the cardiac NCCs’ development.

In summary, GSEA and network analysis of the *Carm1^KO/KO^* transcriptome reveal perturbation of genes involved in the development of the 3 major populations of CPCs, from the first heart field, second heart field, and cardiac NCCs, supporting a role for *Carm1* in multiple steps during cardiac development.

### Assessment of a functional PAX3 defect in *Carm1* mutant mice

**Fig. 6.**
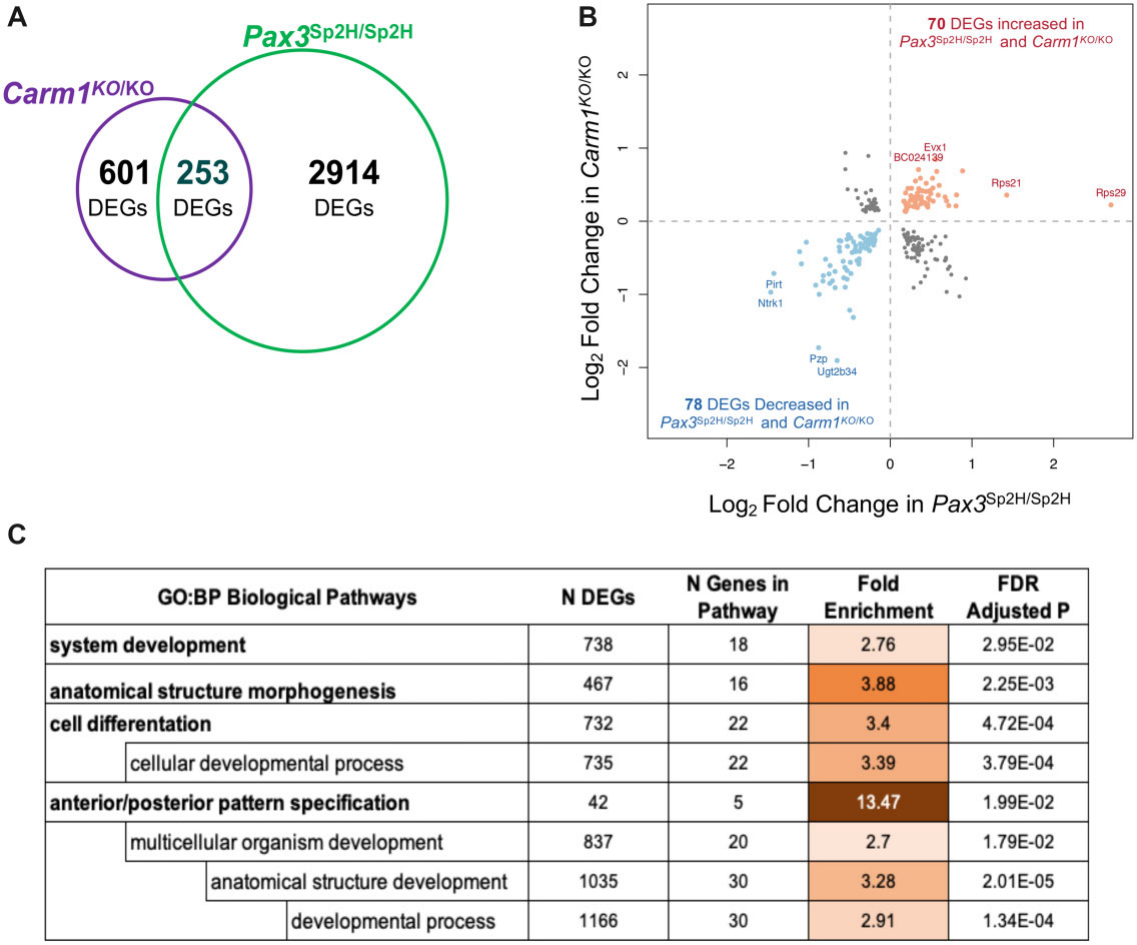
a) RNA sequencing analysis of *Pax3^Sp2H/Sp2H^* and wild-type embryos at E12.5 reveals 3,174 differentially expressed genes (DEGs; *n* = 3WT and 3KO, *P*_adj_ < 5%). b) Of the 254 that are also differentially expressed between *Carm1^KO/KO^* and wild-type embryos, 148 are concordant; i.e. show a log2fold change of expression in the same direction, and 106 are discordant. c) GSEA of the concordant genes reveals enrichment in pathways involved in organ development

The cardiac phenotype of *Carm1* mutant mice is similar to that reported for Splotch (*Pax3^Sp2h/Sp2h^*) mice, which carry a null mutation of *Pax3* (Epstein *et al.* 1991; Conway *et al.* 1997). In vitro studies suggest that PAX3 protein is methylated by CARM1 (Kawabe *et al.* 2012). Given this, we examined whether the defect of cardiac development we see in *Carm1* null mice is primarily due to a functional defect in *Pax3* activity by comparing changes in gene expression in the two mutants. RNA sequencing analysis of 3 *Pax3^Sp2H/Sp2H^* and 3 wild-type embryos at E12.5 reveals 3,167 differentially expressed genes (FDR adjusted *P* < 0.05). Of those genes, 253 are also differentially expressed between *Carm1^KO/KO^* and wild-type embryos (Fig. 6a). One hundred and forty-eight of the differentially expressed genes are concordant; i.e. show a log2fold change of expression in the same direction (70 increased in both, 78 decreased in both), and 105 differentially expressed genes were discordant (Fig. 6b). Of note, in a GSEA analysis, the only pathways in which the 148 concordant genes were significantly overrepresented relate to organ development, including pathways involving anatomical structure morphogenesis, cell differentiation, and anterior/posterior pattern specification (FDR adjusted *P* < 0.05, fishers exact test; Fig. 6c).

### Analysis of CARM1 in congenital heart disease

We examined whole-genome sequence data from 2 large studies of patients with CHDs. The National Heart, Lung, and Blood Institute (NHLBI) Bench to Bassinet Program: The Gabriella Miller Kids First Pediatric Research Program of the Pediatric Cardiac Genetics Consortium (PCGC; phs001138.v3.p2) is accessible in dbGAP and contains 704 affected individuals in trios and NHLBI TOPMed: PCGC’s Congenital Heart Disease Biobank (phs001735.v2.p1) contains a total of 1,072 affected individuals. No de novo mutations in CARM1 were identified, and only one of the missense substitutions was predicted to be damaging (Table 2). Perhaps of interest, both studies had multiple families who carried an in-frame deletion in the N-terminal of the protein (dbSnp rsID: rs763660570). This region is conserved in primates (but not beyond) and this variant is very rare in an unselected population (0.000272439 allele frequency in gnomAD, with a cadd_phred score of 17.4700). It is also identified as a “promoter-like” Cis-Regulatory Element in ENCODE (Accession: EH38E1938728).

**Table 2.** Analysis of DNA sequence variants in patients and families with CHDs.

	Inheritance	Chr	Start	End	Ref	Alt	Func. refGene	ExonicFunc.refGene	AAChange	Polyphen2_ HDIV_pred	Polyphen2_ HVAR_pred	CADD_phred
**phs001735.v2.p1**												
NWD475652	Inherited	19	10871737	10871760	CGGGCGGCGCGGG GTCGGCGGTCC	–	Exonic	Nonframeshift deletion	Exon1: c.35_58del: p.S17_G24del	.	.	.
NWD947532	Inherited	19	10871737	10871760	CGGGCGGCGCGGG GTCGGCGGTCC	–	Exonic	Nonframeshift deletion	Exon1: c.35_58del: p.S17_G24del	.	.	.
NWD645239	Inherited	19	10871743	10871743	G	A	Exonic	Nonsynonymous SNV	Exon1: c.G41A: p.G14D	B	B	17.58
NWD849039	Inherited	19	10871760	10871760	C	T	Exonic	Nonsynonymous SNV	Exon1: c.C58T: p.P20S	B	B	11.49
NWD933034	Inherited	19	10909148	10909148	G	A	Exonic	Nonsynonymous SNV	Exon4: c.G499A: p.V167M	D	D	28
NWD328041	Inherited	19	10920896	10920896	T	C	Exonic	Nonsynonymous SNV	Exon13: c.T1487C: p.M496T	B	B	22.4
**phs001138.v3.p2**												
BS_GQZ0KKFM	Inherited	19	10871736		GCGGGCGGCGCGG GGTCGGCGGTCC: G	–	Exonic	Nonframeshift deletion	Exon1: c.35_58del: p.S17_G24del	.	.	.
BS_J07ETP95	Inherited	19	10871736		GCGGGCGGCGCGG GGTCGGCGGTCC: G	–	Exonic	Nonframeshift deletion	Exon1: c.35_58del: p.S17_G24del	.	.	.
BS_C2EEW8Z4	Inherited	19	10921407		C	T	Exonic	Nonsynonymous SNV	Exon15: c.C1648T: p.R550W	B	B	24.5

## Discussion

### *Carm1* is necessary for heart development

In an ENU mutagenesis screen for neurodevelopmental defects, we identified a line of mice with cortical heterotopias that carried a missense mutation in *Carm1*. Characterization of this line at midgestation revealed severe but incompletely penetrant cardiac defects. Further characterization at late gestation revealed that many of the mutant mice that survived to E18.5 also had cardiac anomalies (VSDs and DORV). The causal nature of the missense mutation we discovered was proven by a complementation test with a *Carm1* null mutant. CARM1 is part of the PRMTs family, which is made up of 10 known enzymes that methylate arginine residues. *Carm1*, *Prmt1*, and *Prmt5* have been implicated in heart function in adult and juvenile mice (Chen *et al.* 2014; Li *et al.* 2017; Murata *et al.* 2018; Pyun *et al.* 2018; Wang *et al.* 2019); however, arginine methyltransferases have not been implicated thus far in cardiac development.

The null allele of *the Carm1^KO^* mouse is a deletion of exons 2 and 3 that encode a protein domain involved in cofactor binding (Yadav *et al.* 2003). Bedford and colleagues did not describe cardiac defects in their characterization of *Carm1^KO^*; however, they did observe a significant reduction of mutant mice relative to expectation at E18.5 (but not at midgestation; Yadav *et al.* 2003).

The ENU-induced A296E missense mutation affects a highly conserved region of the catalytic domain of the protein. Bedford and colleagues directly assessed the role of the CARM1 catalytic domain for in vivo function by generating a mutant mouse line carrying a missense mutation of *Carm1* (*Carm1^R169A^*), a mutant allele that lacks methyltransferase activity in vitro (Kim *et al.* 2010). *Carm1^R169A/R169A^* and *Carm1^KO/KO^* embryos show similar phenotypes, including late embryonic lethality, and we would predict *Carm1^R169A/R169A^* embryos to show similar heart developmental defects.

In humans, CARM1 is intolerant to loss-of-function mutations and has a pLI of 1 (30 loss-of-function mutations expected in the GnomAD v.2.1.1 population of 141,456 samples and none observed; Karczewski *et al.* 2020). This is also evident in our previous analysis of gene essentiality, in which CARM1 falls in the second percentile of 15,998 genes ranked for heterozygote selection (Cassa *et al.* 2017). Our analysis of patient cohorts with CHDs identified a small number with rare, potentially damaging variants, but does not support a major role for CARM1 mutation as a primary cause of this disorder.

### Analysis of the role of PAX3 in *Carm1* mutant mice

CARM1 has a multitude of known targets, including a number known to mediate cardiac development, as discussed below. As such, the causation of the heart defects in *Carm1* mutant mice is likely to be complex. Of particular interest, however, is PAX3. CARM1 regulation of its homolog PAX7 has been characterized in detail (Kawabe *et al.* 2012). The sites of arginine methylation in PAX7 are highly conserved in PAX3 and PAX3 methylation in vitro by CARM1 was shown in the same study. Methylation of PAX3 has been shown to be necessary for its ability to bind mitotic chromosomes (Wu *et al.* 2015). This suggests that PAX3 transcriptional activity mediated by direct DNA binding could be regulated by methylation. Methylation status might also affect its binding with cofactors necessary to activate the transcription of target genes. For instance, MLL2, a histone-lysine N-methyltransferase is recruited by PAX7 to activate the transcription of its target genes, and their interaction is regulated by the methylation of PAX7 by CARM1 (Kawabe *et al.* 2012).

It is furthermore notable that *Pax3* null mutant mice have a cardiac defect that is very similar to *Carm1* null mutants. In both lines, there is marked midgestation lethality, mutants that survive to late gestation have VSDs or DORV, and perinatal lethality is fully penetrant (Conway *et al.* 1997). To explore this similarity in more detail, we examined molecular markers of cardiac development. Most notably, we found that in *Carm1^KO/KO^* embryos, cardiac NCCs expressing *Sema3c* fail to symmetrically condense in the OFT. Lineage tracing also showed that cardiac NCCs can migrate to the OFT in *Pax3* null mutants, but the cells fail to position properly (Conway *et al.* 1997; Epstein *et al.* 2000). Since cells deriving from the cardiac NCCs and expressing *Sema3c* are present in the OFT of *Carm1^KO/KO^* embryos at E12.5 but are mislocalized, we suggest that *Carm1* is not involved in premigratory and migratory cardiac NCCs but in their positioning in the OFT. Failure of cardiac NCCs to position correctly likely results in impaired remodeling and septation of the OFT in the mutants, ultimately causing PTA, DORV, or VSD. Indeed, the OFT septum attaches to the interventricular septum, connecting the base of the aorta and the base of the pulmonary artery to the ventricles (Plein *et al.* 2015).

While the similarity of *Carm1* and *Pax3* null mutant embryos is intriguing, they are not identical, and *Carm1* has many known protein interactions that likely influence the mutant phenotype. Of note, *Carm1^KO/KO^* homozygotes rarely have neural tube defects, which are frequent in *Pax3* null homozygous mice, and *Carm1^KO/+^* mice do not show white belly spots as *Splotch* heterozygous mutants do. Also, while our transcriptional analysis showed a great deal of concordance in differentially expressed genes at E12.5, there was abundant discordance as well. Thus our data do not support the hypothesis that the cardiac defect in *Carm1* mutant mice is primarily a functional defect in *Pax3* activity. This could be genetically tested by an epistasis analysis of doubly mutant mice; however, the intrinsic variability of the single mutant phenotypes could confound the interpretation of this experiment.

A wide variety of proteins have been identified as targets for CARM1 arginine methyltransferase activity. CARM1 has also been identified as having nontranscriptional activator roles (reviewed in Suresh *et al.* 2021). While some of these can be excluded based on the evidence that *Carm1* mutants lacking catalytic activity have the same phenotype as null mutant mice (including their deficiency at late embryogenesis timepoints; Kim *et al.* 2010), the multitude of CARM1 interactions suggests its role in cardiac development is complex. For example, *Mef2c* is a transcription factor that is bilaterally expressed in the heart field and is necessary for heart tube development. Deletion of *Mef2c* is shown to cause embryonic lethality, lack of endocardial cushions, absent right ventricle, lack of elongation of the AV canal, and absent sinus venosus (Lin *et al.* 1997). Conditional deletion of *Mef2c* in the anterior second heart field results in embryos with DORV, tetralogy of Fallot, and transposition of the great arteries (Barnes *et al.* 2016). CARM1 physically interacts with MEF2C and is a transcriptional coactivator; inhibition of CARM1 in myogenic cells abrogated *Mef2c* expression and inhibited differentiation (Chen *et al.* 2002). Of note, the expression of *Mef2c* is significantly reduced in our analysis of differential expression.

CARM1 interacts with several other genes that are required for normal heart development. CARM1 methylates the NICD-coactivator complex of NOTCH1 (Hein *et al.* 2015), which is part of a known pathway for EMT and endocardium development. Null mutants of *Notch1* exhibit Tetralogy of Fallot, pulmonary stenosis, atrio-VSD, coarctation of the aorta, hyperplastic left heart syndrome, and left ventricular OFT malformations (Swiatek *et al.* 1994; Conlon *et al.* 1995; Timmerman *et al.* 2004; Grego-Bessa *et al.* 2007). *Sox9* is a well-characterized transcription factor that is methylated by CARM1 at its HMG domain, disrupting its interaction with β-catenin (Ito *et al.* 2009). A decrease in Wnt/β-catenin leads to overexpression of *Sox9*, excessive proteoglycan accumulation, and disruption of the extracellular matrix in valve leaflets, causing myxomatous valve disease. The phenotype of *Sox9* null mutants includes dilated blood vessels, hypoplasia of frontonasal mesenchyme, otic vesicles, branchial arches, as well as endocardial cushion hypoplasia (Akiyama *et al.* 2004); this latter defect is similar to that found in the *Carm1* null mutants.

### Methylation and cardiac development

An emerging theme in the genetic analysis of CHD is the importance of mutations in genes involved in the regulation of chromatin (Chang and Bruneau 2012; Zaidi *et al.* 2013). It is well-established that histones are a target of CARM1 methylation activity (Schurter *et al.* 2001). In addition, CARM1 interacts with other proteins involved in chromatin modification such as p300/CREB-binding protein (An *et al.* 2004). Notably, while there are a large number of chromatin-modifying genes that have been implicated in CHD, the number of mutations in patients for most of these genes individually is small. Furthermore, most of these are normally widely expressed, such that their functional absence in patients and mouse mutants affects many proteins and is not generally confined to the developing heart. It seems likely that the mammalian heart is simply the most sensitive organ to epigenetic dysregulation during organogenesis, which would explain why the investigation of genetic causality in this disorder has been so challenging.

## Data availability

The mouse line *Carm1^A296E^* is available upon request and will be deposited with the Mouse Mutant Resource and Research Center repository. Gene expression data are available at GEO with the accession number: GSE193588.

Supplemental material is available at *G3* online.

## Supplementary Material

jkac155_Supplemental_Material_legends

jkac155_Figure_S1

jkac155_Figure_S2

jkac155_Figure_S3

jkac155_Figure_S4

jkac155_Figure_S5

jkac155_Table_S1

jkac155_Table_S2
