# Supplementary material for: The arginine methyltransferase Carm1 is necessary for heart development
Source: G3 (Bethesda). 2022 Jun 23;12(8):jkac155. doi: 10.1093/g3journal/jkac155 (PMC9339313; doi:10.1093/g3journal/jkac155)
Supplement: jkac155_Supplemental_Material_legends [file jkac155_supplemental_material_legends.docx]

Figure S1: The domain of CARM1 that is mutated in the *Carm1^A296E^* line is very highly conserved. AA 296 is highlighted.

Fig. S2: A) Complementation test. The double heterozygote *Carm1^A296E/KO^* mutant shows the same phenotype as *Carm1^A296E^* homozygotes: smaller embryo size, omphalocele, abnormal limb morphology, abnormal craniofacial morphology, and cleft palate. B) An E12.5 *Carm1^KO^* mutant embryo with blood in the thoracic cavity.

Fig. S3: Cardiac defects in *Carm1^KO^* mutants. A) Histology of an E12.5 *Carm1^KO^* mutant embryo showing persistent truncus arteriosus. Ao, aorta; PA, pulmonary artery. B) Histology of an E18.5 *Carm1^KO^* mutant embryo showing a ventricular septal defect (arrow). C) Histology of an E18.5 *Carm1^KO^* mutant embryo showing double-outlet right ventricle.

Figure S4: MicroCT analysis of an E18.5 *Carm1^A296E^* mutant embryo showing a ventricular septal defect (arrow).

Figure S5: A) tissue specific expression of *Carm1* during embryonic development (from Mouse Organogenesis Cell Atlas (Cao et al. 2019)). B) whole mount *in situ* hybridization analysis of *Carm1* at E10.5. C) analysis of single-cell *Carm1* expression in cardiac development (from the Early Mouse Cardiogenesis project (de Soysa et al. 2019)). *Carm1-* expressing cells are beige.

Table S1: GSEA reveals 94 pathways enriched for genes whose expression was significantly decreased in *Carm1^KO/KO^* vs. wild-type mice.

Table S2: Targeted pathway analysis to examine differentially expressed genes in *Carm1^KO/KO^* vs. wild-type mice known to be involved in heart development. Genes are listed according to FDR adjusted p-value.
