# Supplementary figures and images for: The arginine methyltransferase Carm1 is necessary for heart development
Source: G3 (Bethesda). 2022 Jun 23;12(8):jkac155. doi: 10.1093/g3journal/jkac155 (PMC9339313; doi:10.1093/g3journal/jkac155)

## Figure S1

# A296E

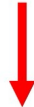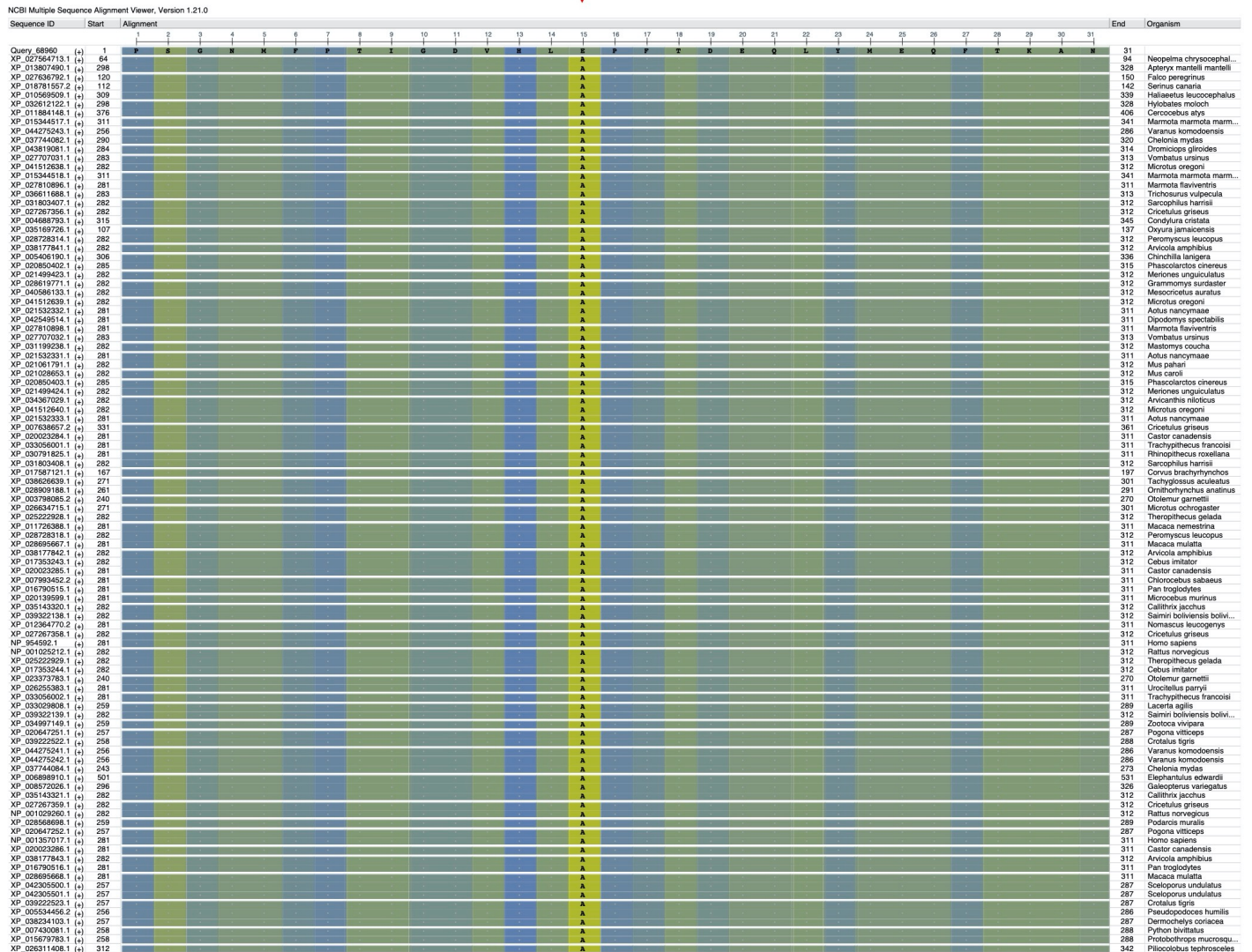

Supplement: jkac155_Figure_S1 [file jkac155_figure_s1.pdf]

Figure S2

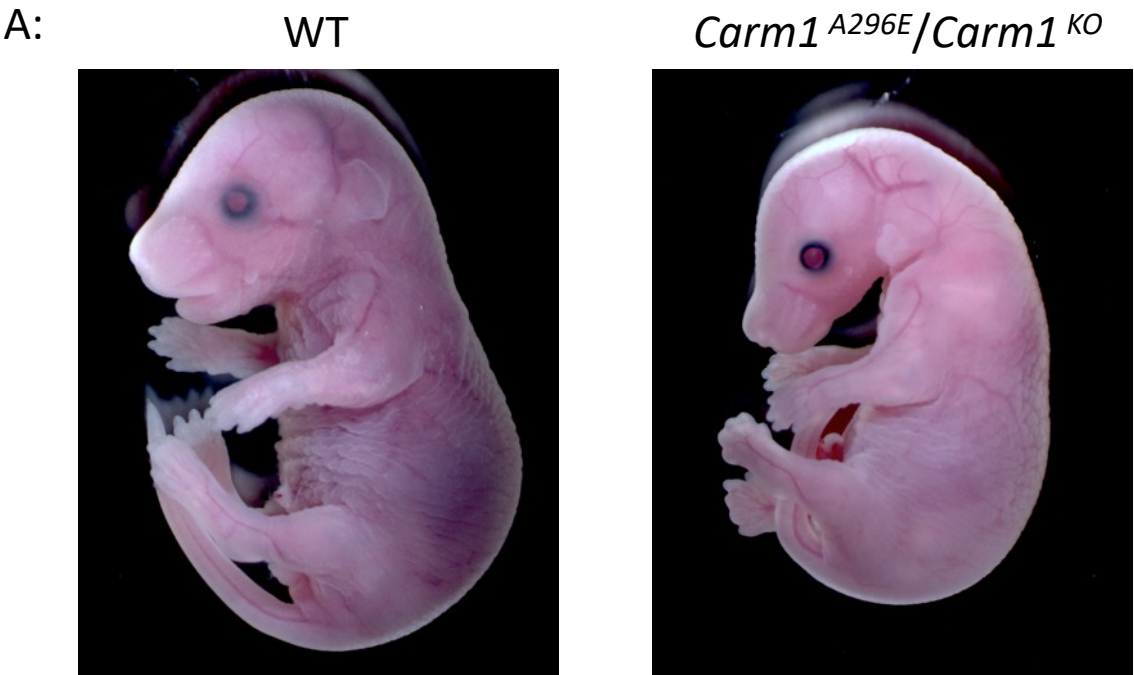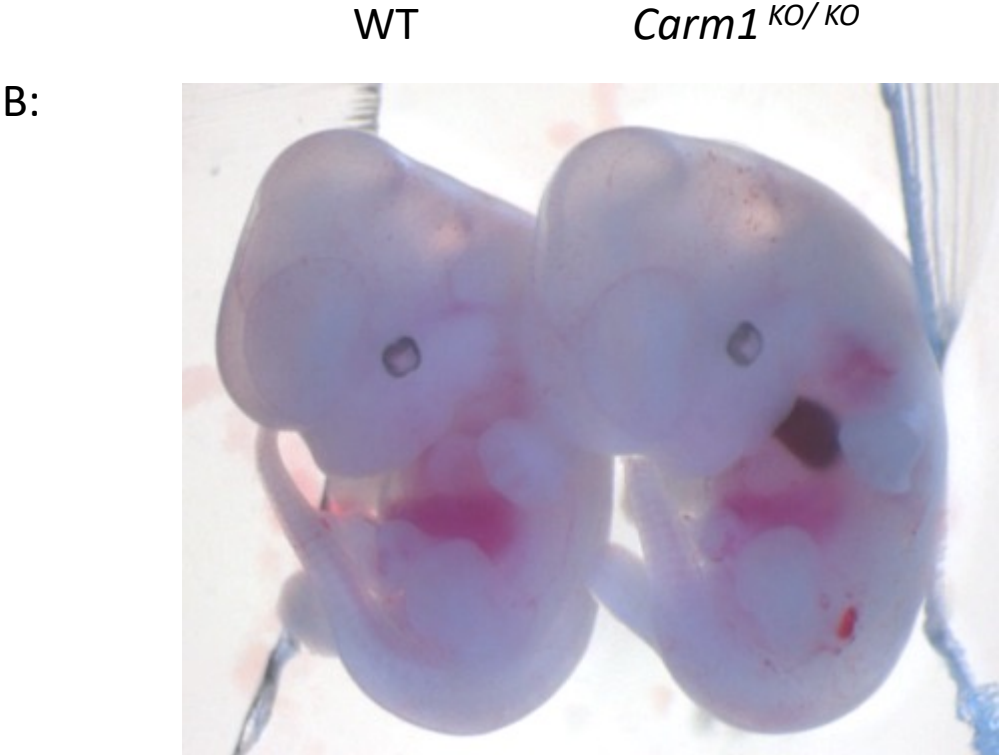

Supplement: jkac155_Figure_S2 [file jkac155_figure_s2.pdf]

Figure S4

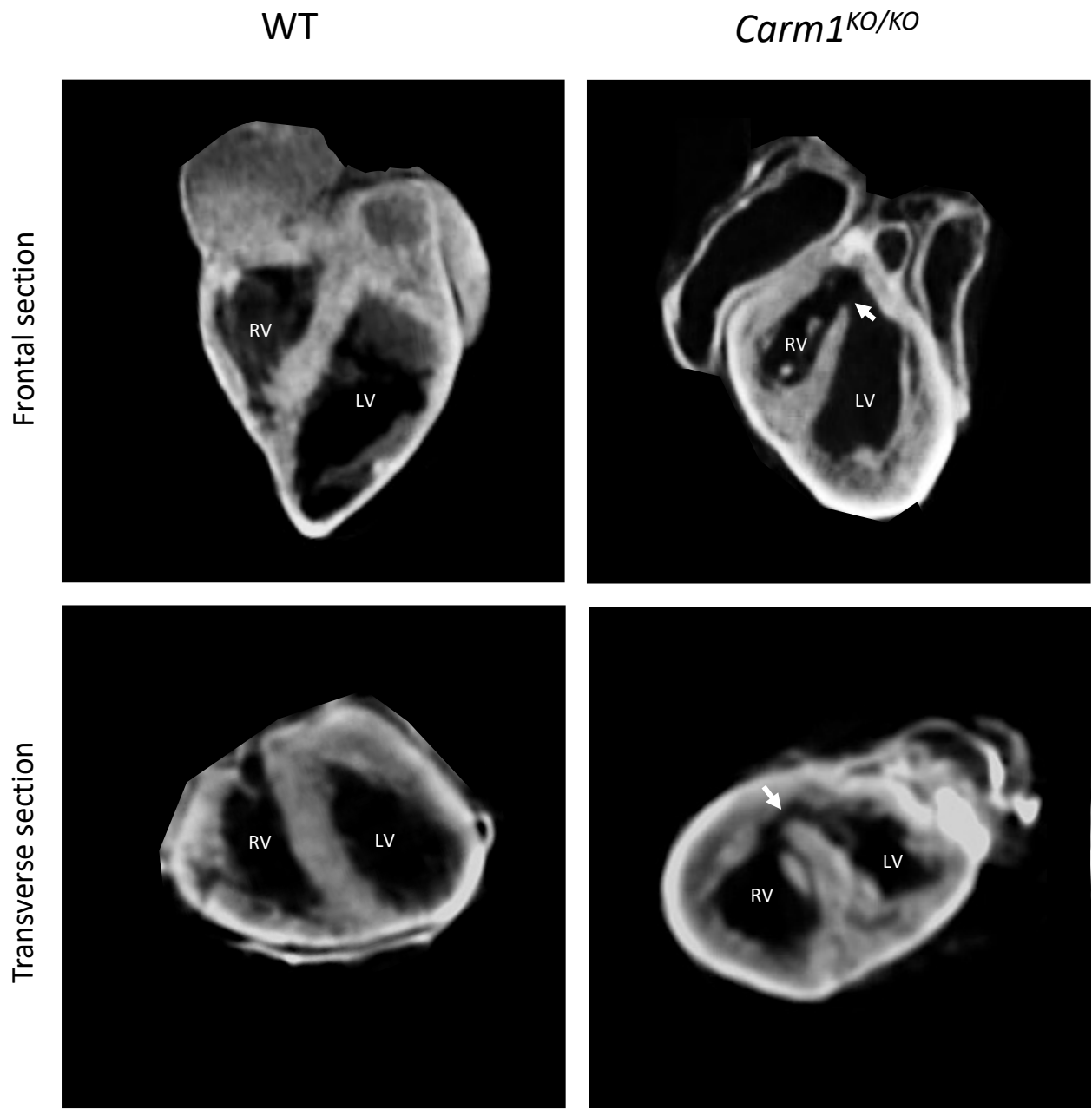

Supplement: jkac155_Figure_S4 [file jkac155_figure_s4.pdf]

Figure S5

A:

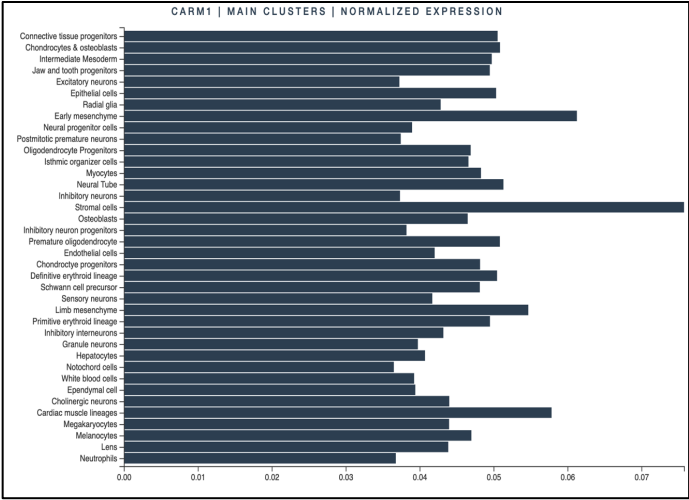

B:

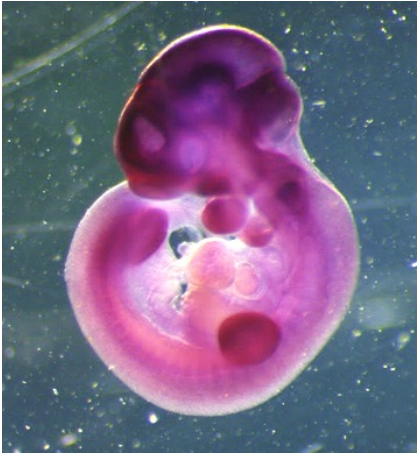

C:

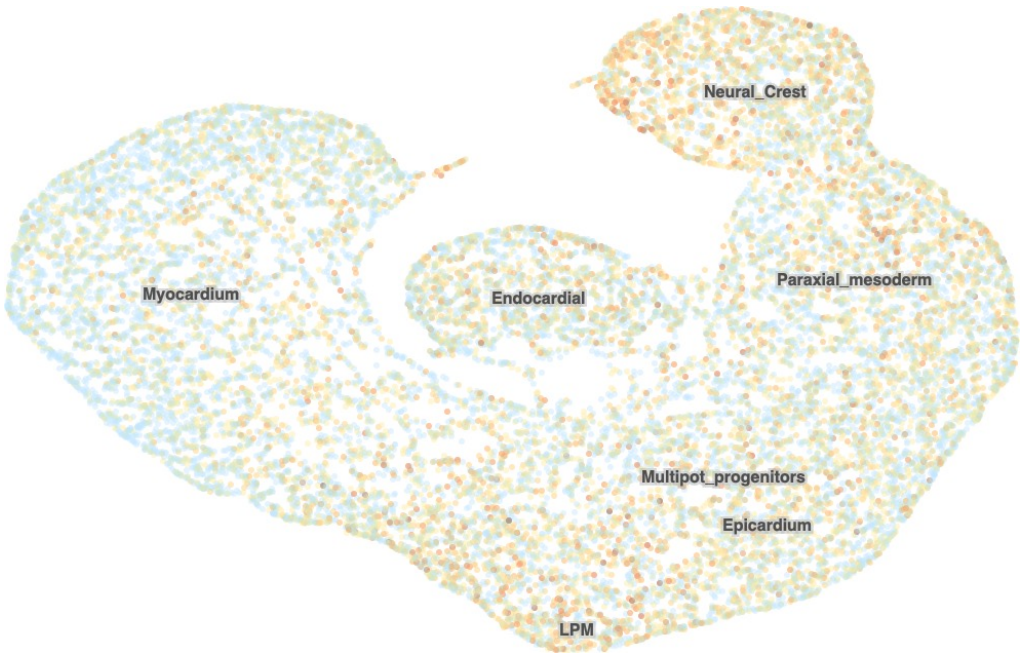

Supplement: jkac155_Figure_S5 [file jkac155_figure_s5.pdf]
