# Supplementary material for: The arginine methyltransferase Carm1 is necessary for heart development
Source: G3 (Bethesda). 2022 Jun 23;12(8):jkac155. doi: 10.1093/g3journal/jkac155 (PMC9339313; doi:10.1093/g3journal/jkac155)
Supplement: jkac155_Figure_S3 [file jkac155_figure_s3.pdf]

Figure S3

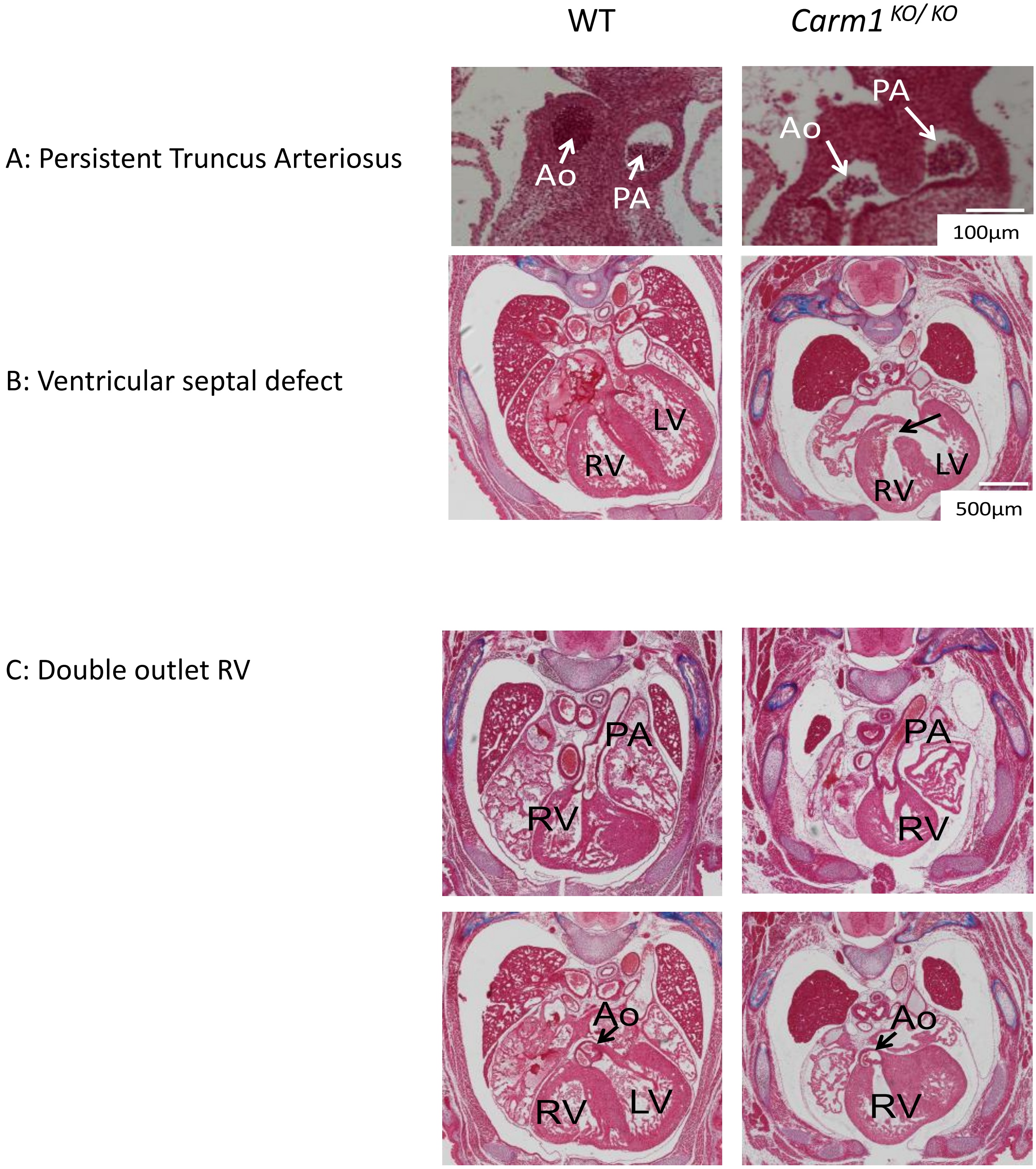
WT
Carm1 KO/ KO
A: Persistent Truncus Arteriosus
Ao
PA
PA
Ao
100μm
B: Ventricular septal defect
LV
RV
LV
RV
500μm
C: Double outlet RV
PA
RV
PA
RV
Ao
RV
LV
Ao
RV
