# Supplementary material for: The arginine methyltransferase Carm1 is necessary for heart development
Source: G3 (Bethesda). 2022 Jun 23;12(8):jkac155. doi: 10.1093/g3journal/jkac155 (PMC9339313; doi:10.1093/g3journal/jkac155)
Supplement: jkac155_Table_S1 [file jkac155_table_s1.pdf]

SUPPLEMENTAL TABLE 1

GO:BP Pathway	Go Term	Pathway Size	Enrichemnt S	Normalized I	NOM p-val	FDR q-val
NEGATIVE REGULATION OF COAGULATION	GO:0030195	51	-0.7735631	-2.3508918	0	0
REGULATION OF COAGULATION	GO:0050818	70	-0.7287689	-2.3439517	0	0
FIBRINOLYSIS	GO:0042730	25	-0.8626722	-2.297576	0	0
NEGATIVE REGULATION OF WOUND HEALING	GO:0061045	69	-0.6735635	-2.1891453	0	0
PROTEIN ACTIVATION CASCADE	GO:0072376	28	-0.7918164	-2.1752913	0	1.79E-04
MUSCLE FILAMENT SLIDING	GO:0030049	39	-0.7052714	-2.0819914	0	5.91E-04
PLASMINOGEN ACTIVATION	GO:0031639	22	-0.805794	-2.0764773	0	6.32E-04
COMPLEMENT ACTIVATION	GO:0006956	59	-0.6721631	-2.0744395	0	5.53E-04
POSITIVE REGULATION OF COAGULATION	GO:0050820	24	-0.7724443	-2.064634	0	5.90E-04
HOMOPHILIC CELL ADHESION VIA PLASMA MEMBRANE ADHESION MOLECULES	GO:0007156	156	-0.5760297	-2.063997	0	5.31E-04
REGULATION OF COMPLEMENT ACTIVATION	GO:0030449	45	-0.7003301	-2.0636458	0	4.83E-04
GLUTAMATE METABOLIC PROCESS	GO:0006536	31	-0.7316627	-2.0421033	0	0.00110522
STEROL HOMEOSTASIS	GO:0055092	86	-0.6134646	-2.0415916	0	0.0010202
SKELETAL MUSCLE CONTRACTION	GO:0003009	43	-0.6767889	-2.024437	0	0.00113749
STRIATED MUSCLE CELL DEVELOPMENT	GO:0055002	103	-0.5951109	-2.0133033	0	0.00147784
NEGATIVE REGULATION OF RESPONSE TO WOUNDING	GO:1903035	85	-0.6076316	-1.9964089	0	0.00216002
PLATELET DEGRANULATION	GO:0002576	126	-0.5798584	-1.9916025	0	0.00229311
BLOOD COAGULATION INTRINSIC PATHWAY	GO:0007597	18	-0.7909905	-1.9881787	0	0.00231392
RESPONSE TO XENOBIOTIC STIMULUS	GO:0009410	101	-0.5906527	-1.986189	0	0.00223888
ALPHA AMINO ACID METABOLIC PROCESS	GO:1901605	181	-0.5531925	-1.9791517	0	0.00252506
HIGH DENSITY LIPOPROTEIN PARTICLE REMODELING	GO:0034375	17	-0.7913914	-1.9747194	0	0.00261491
HUMORAL IMMUNE RESPONSE MEDIATED BY CIRCULATING IMMUNOGLOBULIN	GO:0002455	44	-0.6697238	-1.9715319	0	0.00257638
REGULATION OF WOUND HEALING	GO:0061041	124	-0.5648311	-1.9626619	0	0.00300506
ZYMOGEN ACTIVATION	GO:0031638	54	-0.6304064	-1.9620951	0	0.00299138
REVERSE CHOLESTEROL TRANSPORT	GO:0043691	17	-0.7941126	-1.9515073	0	0.00361377
MYOFIBRIL ASSEMBLY	GO:0030239	59	-0.6263283	-1.9441757	0	0.00422298
CELLULAR AMINO ACID CATABOLIC PROCESS	GO:0009063	101	-0.5704007	-1.9365698	0	0.00449348
CELLULAR MODIFIED AMINO ACID METABOLIC PROCESS	GO:0006575	176	-0.5402543	-1.935388	0	0.00439665
ALPHA AMINO ACID CATABOLIC PROCESS	GO:1901606	84	-0.5772709	-1.9092675	0	0.00730517
MULTICELLULAR ORGANISMAL MOVEMENT	GO:0050879	54	-0.6293265	-1.9025308	0	0.00833306
TRIGLYCERIDE METABOLIC PROCESS	GO:0006641	97	-0.5629927	-1.9017866	0	0.00826471
LIPID HOMEOSTASIS	GO:0055088	149	-0.5374376	-1.8984987	0	0.00842116
REGULATION OF HUMORAL IMMUNE RESPONSE	GO:0002920	66	-0.5922858	-1.8983151	0	0.00824588
MUSCLE FIBER DEVELOPMENT	GO:0048741	53	-0.6232404	-1.8944305	0	0.00849867
POSITIVE REGULATION OF ACUTE INFLAMMATORY RESPONSE	GO:0002675	26	-0.6978191	-1.8906919	0.00158479	0.00871308
SARCOMERE ORGANIZATION	GO:0045214	38	-0.6551114	-1.8836745	0	0.00950799
NEUTRAL LIPID METABOLIC PROCESS	GO:0006638	124	-0.5397179	-1.8832161	0	0.00939545
HUMORAL IMMUNE RESPONSE	GO:0006959	181	-0.515868	-1.8693714	0	0.01220725
PROTEIN LIPID COMPLEX SUBUNIT ORGANIZATION	GO:0071825	51	-0.6164494	-1.8675641	0	0.01228169
REGULATION OF HETEROTYPIC CELL CELL ADHESION	GO:0034114	23	-0.7159684	-1.8649069	0	0.01265962
ACYLGLYCEROL HOMEOSTASIS	GO:0055090	36	-0.6526229	-1.8622624	0	0.01295808
CELLULAR COMPONENT ASSEMBLY INVOLVED IN MORPHOGENESIS	GO:0010927	98	-0.5477317	-1.8498975	0	0.01600609
DRUG METABOLIC PROCESS	GO:0006805	35	-0.6482729	-1.842183	0	0.01779327
NEUTRAL LIPID CATABOLIC PROCESS	GO:0046461	44	-0.6173078	-1.8355348	0	0.01936084
CELL CELL ADHESION VIA PLASMA MEMBRANE ADHESION MOLECULES	GO:0098742	255	-0.493064	-1.8349031	0	0.019148
PROTEIN CONTAINING COMPLEX REMODELING	GO:0034367	31	-0.6611991	-1.8331667	0	0.01934716
NEGATIVE REGULATION OF LIPASE ACTIVITY	GO:0060192	15	-0.7832933	-1.8255211	0.00162866	0.02127591
THYROID HORMONE METABOLIC PROCESS	GO:0042403	25	-0.6847867	-1.8222182	0.0015674	0.02216156
PROTEIN LIPID COMPLEX ASSEMBLY	GO:0065005	34	-0.6498909	-1.8199507	0	0.02255846
POSITIVE REGULATION OF LIPID LOCALIZATION	GO:1905954	107	-0.5387834	-1.8051623	0	0.02770918
REGULATION OF RESPONSE TO WOUNDING	GO:1903034	153	-0.5090975	-1.8009099	0	0.02925374
REGULATION OF TRIGLYCERIDE METABOLIC PROCESS	GO:0090207	36	-0.6183868	-1.7995123	0	0.02915081
GLUTAMINE FAMILY AMINO ACID METABOLIC PROCESS	GO:0009064	70	-0.5607608	-1.7968545	0	0.02978757
CYTOLYSIS	GO:0019835	22	-0.6838293	-1.7961001	0	0.029466
ALPHA AMINO ACID BIOSYNTHETIC PROCESS	GO:1901607	62	-0.5679619	-1.7949932	0	0.02938115
PLASMA LIPOPROTEIN PARTICLE CLEARANCE	GO:0034381	60	-0.5727426	-1.7901734	0	0.03145293
CORNIFICATION	GO:0070268	80	-0.5452133	-1.7900254	0.00135501	0.03102608
CELLULAR MODIFIED AMINO ACID CATABOLIC PROCESS	GO:0042219	26	-0.6616153	-1.787964	0.00156006	0.03160684
ORGANIC HYDROXY COMPOUND TRANSPORT	GO:0015850	252	-0.4796192	-1.7868927	0	0.03159867
STEROID ESTERIFICATION	GO:0034433	18	-0.7211016	-1.783472	0.0016129	0.03289208
REGULATION OF LIPID LOCALIZATION	GO:1905952	176	-0.4961075	-1.7789074	0	0.03455088
BIOLOGICAL PROCESS INVOLVED IN INTRASPECIES INTERACTION BETWEEN ORGANISM	GO:0051703	49	-0.5893423	-1.7757965	0	0.03553958
MUSCLE CELL DEVELOPMENT	GO:0055001	163	-0.4963969	-1.7750314	0	0.03528562
GLYCEROLIPID CATABOLIC PROCESS	GO:0046503	70	-0.5542023	-1.7739131	0.00135685	0.0353721
DICARBOXYLIC ACID CATABOLIC PROCESS	GO:0043649	15	-0.7278737	-1.7733959	0.0017301	0.03516838
METHIONINE METABOLIC PROCESS	GO:0006555	16	-0.7490352	-1.7677337	0	0.03764478
CELLULAR AMINO ACID METABOLIC PROCESS	GO:0006520	318	-0.464368	-1.7611738	0	0.04096109
APOPTOTIC CELL CLEARANCE	GO:0043277	48	-0.5853427	-1.7608365	0.00144928	0.04051514
POSITIVE REGULATION OF SUBSTRATE ADHESION DEPENDENT CELL SPREADING	GO:1900026	41	-0.5977889	-1.7589374	0.0014556	0.04098458
HIGH DENSITY LIPOPROTEIN PARTICLE ASSEMBLY	GO:0034380	15	-0.7427153	-1.7585578	0.00318979	0.04061484
NEURONAL ACTION POTENTIAL	GO:0019228	32	-0.6265432	-1.7572181	0	0.04089288
NEGATIVE REGULATION OF ATPASE ACTIVITY	GO:0032780	17	-0.7152149	-1.7562572	0.0032	0.0410276
CELLULAR AMINO ACID BIOSYNTHETIC PROCESS	GO:0008652	71	-0.5504699	-1.7562431	0	0.04046558
EXPLORATION BEHAVIOR	GO:0035640	19	-0.6939862	-1.7549536	0.00325203	0.04070878
XENOBIOTIC TRANSPORT	GO:0042908	38	-0.6123098	-1.7536461	0.00151745	0.04095955
SYNAPSE ASSEMBLY	GO:0007416	168	-0.4843618	-1.7478087	0	0.04393112
ORGANIC ACID BIOSYNTHETIC PROCESS	GO:0016053	305	-0.4605887	-1.7468085	0	0.04396022
KILLING OF CELLS IN OTHER ORGANISM INVOLVED IN SYMBIOTIC INTERACTION	GO:0051883	17	-0.707116	-1.7467033	0.00166389	0.04345335
TERPENOID METABOLIC PROCESS	GO:0006721	102	-0.5168538	-1.7464498	0	0.04307217
STRIATED MUSCLE CONTRACTION	GO:0006941	169	-0.4878318	-1.7461164	0	0.04273351
AMINE CATABOLIC PROCESS	GO:0009310	27	-0.632896	-1.7454423	0.00157978	0.04269818
REGULATION OF HYDROGEN PEROXIDE METABOLIC PROCESS	GO:0010310	17	-0.7135719	-1.7425337	0.005	0.04405788
POSITIVE REGULATION OF LIPID STORAGE	GO:0010884	27	-0.6453933	-1.7404639	0.0031746	0.04473352
PRIMARY ALCOHOL METABOLIC PROCESS	GO:0034308	72	-0.5420957	-1.7397541	0.00280899	0.0447086
SULFUR AMINO ACID BIOSYNTHETIC PROCESS	GO:0000097	16	-0.7149745	-1.7393774	0.00323102	0.0444849
REGULATION OF PLASMA LIPOPROTEIN PARTICLE LEVELS	GO:0097006	90	-0.5215989	-1.7351092	0	0.04631716
LIPID LOCALIZATION	GO:0010876	479	-0.4514456	-1.7347987	0	0.04604954
DICARBOXYLIC ACID METABOLIC PROCESS	GO:0043648	92	-0.5124106	-1.7336959	0	0.04625261
REGULATION OF LIPID TRANSPORT	GO:0032368	144	-0.4901537	-1.7320037	0	0.04683094
NEGATIVE REGULATION OF CELLULAR RESPONSE TO VASCULAR ENDOTHELIAL GROWT	GO:1902548	15	-0.7255405	-1.7290701	0.00815661	0.04836074
POSITIVE REGULATION OF FATTY ACID METABOLIC PROCESS	GO:0045923	38	-0.5913231	-1.7288173	0.00147059	0.0479852
STEROID METABOLIC PROCESS	GO:0008202	290	-0.458539	-1.727406	0	0.04834085
ACTIN MEDIATED CELL CONTRACTION	GO:0070252	118	-0.4929826	-1.7263825	0	0.04845079
TRIGLYCERIDE CATABOLIC PROCESS	GO:0019433	34	-0.6046506	-1.7239693	0	0.04952136
