# Supplementary material for: The arginine methyltransferase Carm1 is necessary for heart development
Source: G3 (Bethesda). 2022 Jun 23;12(8):jkac155. doi: 10.1093/g3journal/jkac155 (PMC9339313; doi:10.1093/g3journal/jkac155)
Supplement: jkac155_Table_S2 [file jkac155_table_s2.pdf]

SUPPLEMENTAL TABLE 2

	log2 Fold Change *Carm1* KO vs. WT	FDR Adjusted P
Shh	0.77	2.12E-04
Mef2c	-0.27	3.37E-03
Foxc1	0.30	5.16E-03
Nfatc4	-0.22	6.05E-03
Hand1	0.94	1.55E-02
Smyd1	-0.34	1.96E-02
Nfatc2	-0.57	4.06E-02
Ctnnb1	0.11	4.44E-02
Nfatc1	-0.29	0.09
Fgf8	0.99	0.16
Tbx2	0.17	0.20
Hey1	0.24	0.21
Notch1	0.12	0.35
Erbb3	-0.18	0.43
Irx4	-0.25	0.62
Bmp2	-0.22	0.64
Gata6	0.17	0.67
Foxc2	0.12	0.71
Ptpn11	0.04	0.73
Nkx2-5	0.31	0.74
Vegfc	0.15	0.75
Tbx5	-0.09	0.82
Hey2	0.12	0.82
Nfatc3	0.05	0.83
Vegfb	-0.07	0.85
Mapk1	-0.03	0.85
Foxa2	0.16	0.87
Smad4	0.03	0.87
Bmp4	0.06	0.88
Hand2	0.05	0.89
Foxh1	0.28	0.90
Bhlhe40	-0.11	0.91
Pitx2	0.05	0.91
Vegfa	0.02	0.93
Tbx20	0.05	0.93
Fgf10	-0.06	0.93
Bmpr1a	-0.02	0.94
Srf	-0.02	0.94
Bmp10	0.14	0.95
Smad1	-0.02	0.95
Gata4	-0.03	0.96
Isl1	0.00	0.99
Bmpr2	0.00	1.00
